# Clozapine prescribing in treatment-resistant schizophrenia – an updated systematic literature review of barriers and facilitators among clinicians

**DOI:** 10.1007/s00228-026-04046-2

**Published:** 2026-04-11

**Authors:** Siobhán Nolan, Laura J. Sahm, Ita Fitzgerald

**Affiliations:** 1 Pharmaceutical Care Research Group, School of Pharmacy, University College Cork, Cork, Ireland; 2 Pharmacy Department, St Patrick’s Mental Health Services, Dublin, Ireland; 3 School of Pharmacy, College of Health and Life Sciences, Aston University, Birmingham, UK

**Keywords:** Clozapine, Prescribing, Barriers, Facilitators, Schizophrenia, Systematic review

## Abstract

**Background:**

Increased rates of clozapine prescribing are essential to improving timely patient access within treatment-resistant schizophrenia (TRS) management. The extent of geographical variation in its use suggests it is possible to develop interventions to increase clinician engagement. To inform intervention development, a contemporary review of barriers and facilitators to increased clozapine prescribing is required. We aimed to conduct a systematic review of research addressing barriers and facilitators to clozapine prescribing among clinicians within TRS management.

**Methods:**

The review was conducted in accordance with Preferred Reporting Items for Systematic Reviews and Meta-Analyses (PRISMA) guidelines. PubMed, Embase, CINAHL, and PsycINFO were searched from inception to July 2025. Results were synthesized qualitatively.

**Results:**

Fifteen studies were included. Barriers related to clinicians, patients and carers, and healthcare institutions. Primary clinician-related barriers included insufficient knowledge of, and confidence in, managing clozapine treatment and the associated administrative burden. Primary patient-related barriers included concerns regarding patients’ willingness to consistently adhere to clozapine treatment and associated monitoring requirements. A lack of dedicated systems of care to facilitate clozapine initiation and shared community care were the leading institutional barriers. Major facilitators included improved education for clinicians, access to point-of-care testing, and increased availability of dedicated clozapine clinics.

**Conclusion:**

Most barriers to systematically increasing clozapine prescribing rates are beyond the influence of individual prescribers. Instead, structural interventions focusing on (i) reducing the administrative burden associated with establishing clozapine treatment, (ii) increasing access to standardised training and supervision opportunities, and (iii) providing longitudinal support to clinicians when managing clozapine treatment, are required.

**Supplementary Information:**

The online version contains supplementary material available at 10.1007/s00228-026-04046-2.

## Background

Clozapine remains the only approved medication for managing TRS, a condition impact 30–40% of people diagnosed with schizophrenia [1, 2]. Despite international guidelines recommending clozapine as the most effective treatment in managing TRS [3, 4], it remains significantly underutilised. Research on international prescribing trends indicates that significant delays to clozapine initiation are common [5, 6]. Prompt initiation is crucial as there is evidence of decreased response to clozapine following delayed initiation [7, 8]. Key to increasing timely patient access to clozapine is to increase prescribing rates among clinicians. The extent of geographical variation in use suggests it is possible to develop interventions aimed at increasing clinician engagement.

Previous systematic reviews have found that lack of knowledge regarding the effectiveness of clozapine does not appear as a major determinant of its under-prescription [9]. Given the extent to which knowledge is available regarding the superior efficacy of clozapine within TRS management [2, 4], and the apparent resistance to increased prescribing among clinicians, the interventions required to systematically increase clinician engagement are likely to be complex interventions that require significant behavioural change among clinicians [10]. Developing such interventions requires an understanding of what strategies are (and are not) effective at increasing clinician willingness to prescribe clozapine in a timely manner among people with TRS. Knowledge of effective implementation contexts is also required. The first step in generating this knowledge is to develop a contemporary understanding of barriers and facilitators to increased clozapine prescribing by clinicians. Such barriers and facilitators are likely to be different within different contexts, including among various patient cohorts, and variable among clinicians with different levels of responsibility for the prescribing and management of clozapine treatment [11]. 

We aimed to conduct a systematic review of research addressing barriers and facilitators to clozapine prescribing among clinicians responsible for prescribing clozapine within TRS management. Of note, this could include patient- and service-related barriers, but from the perspective of clinicians only. The most recent systematic review in this area was reflective of searches conducted up to and including 2021 [12], and involved a wide range of clinician views beyond those responsible for prescribing clozapine [9, 12, 13]. More recent studies within the scope of this review have also been published since the last systematic review [14, 15]. 

## Methods

This systematic review was undertaken in compliance with guidance outlined within the Preferred Reporting Items for Systematic Reviews and Meta-Analyses (PRISMA) statement [16]. A copy of the completed PRISMA statement is contained within the supplementary appendix.

### Eligibility criteria

We hypothesized that barriers and facilitators to clozapine prescribing among clinicians would likely be different among different contexts. This includes among people of different ages (e.g., use in adolescents versus older adults) and within different clinical contexts (e.g., TRS management versus Parkinson’s disease). Thus, stringent inclusion and exclusion criteria were developed to reflect the intended scope of the review.

Studies were included where they met the following criteria:

1. Focussed on the perspective of a clinician responsible for the prescribing of clozapine treatment. The term ‘clinician’ is used here to reflect the diverse nature of mental health service delivery in different countries, whereby nurse or pharmacist prescribers, or general practitioners, may facilitate continued prescribing of clozapine within some countries [17]. Thus, studies were eligible where they included views of the clinician responsible for prescribing clozapine, rather than psychiatrists’ views only.
2. Studied prescribing of clozapine in the context of TRS management among adults (aged 18–75 years old).
3. Primary research.
4. Studied outcomes of interest to the review, i.e., barriers and facilitators to clozapine prescribing.
5. Published in a peer-reviewed journal.
6. Available as a full text.
7. Published in English.

Thus, studies with diverse methodologies were eligible where they could use varying methods to collect information on barriers and facilitators to clozapine prescribing among clinicians, including both qualitative and quantitative methods.

Studies were excluded where they:

1. Examined barriers and facilitators to clozapine prescribing, clozapine solely in the context of other psychiatric diagnoses. Where studies addressed clozapine prescribing in TRS alongside other indications, for example, Parkinson’s disease, these were included, but only data relevant to clozapine prescribing in TRS were extracted.
2. Examined patterns or rates of clozapine utilisation exclusively or aimed to determine clozapine’s efficacy or effectiveness in different contexts.
3. Exploring only patient views or perspectives on barriers and facilitators to clozapine prescribing.

There were no restrictions on study setting or year of publication. Grey literature, including unpublished material, was not included, for example, letters, editorials, or commentaries.

### Search strategy

Four electronic databases were searched for eligible studies from inception through 30th of July 2025: PubMed, Embase, CINAHL, and PsycINFO. Search terms related to: (1) psychosis and treatment-resistant schizophrenia, (2) clozapine treatment, (3) prescribers or clinicians, and (4) barriers, facilitators, or impacts on prescribing practices. Synonyms and related truncation were used in combination with Boolean operators across all databases. Database searches were initially conducted in January 2025 and were subsequently repeated in July 2025. A copy of the refined search strategy, using the PubMed database as an example, is contained within the supplementary appendix. Supplementary search methods included searching of reference lists of included studies and prior similar systematic reviews. Alerts for any newer publications were also established via PubMed.

### Study selection

Rayyan (rayyan.ai) systematic review software was used to screen title and abstracts of all studies identified as potentially eligible. Following duplicate removal, two researchers (SN and LS) performed title and abstract screening independently. All studies agreed as being initially eligible for inclusion were then reviewed in full and assessed against prespecified inclusion and exclusion criteria by two researchers independently (SN and LS). Any disagreements were resolved through discussion until consensus was reached. Discussion with a third researcher (IF) was used where needed to achieve consensus.

### Data extraction and synthesis

Data were extracted from included studies according to the aim of this review. Data extraction was initially completed by one researcher (SN) and was subsequently repeated independently by another researcher (IF). Any disagreements were discussed between IF and SN until consensus was reached. Discussion with a third researcher (LS) was available where needed to reach consensus. For studies meeting inclusion criteria, in addition to details of authorship and year of publication, the following details were extracted:

1. Country of study origin.
2. Primary aims and objectives relevant to this systematic review.
3. Study methodology.
4. Study population.
5. Key findings subdivided into primary barriers and facilitators identified.

Due to the diverse and primarily qualitative nature of results reported within eligible studies, results were qualitatively synthesized in accordance with the Cochrane-Campbell Handbook for Qualitative Evidence Synthesis [18]. Findings from the included studies were synthesised using a thematic and iterative approach, utilising an extraction table to organise data into three overarching themes: (1) clinician-related, (2) patient and family-related, and (3) institutional and healthcare system-related barriers. Given the qualitative nature of this review, the frequency with which both barriers and facilitators are described reflects only the breadth of reporting across studies, rather than their relative impact or severity.

## Results

### Study selection

**Fig. 1:**
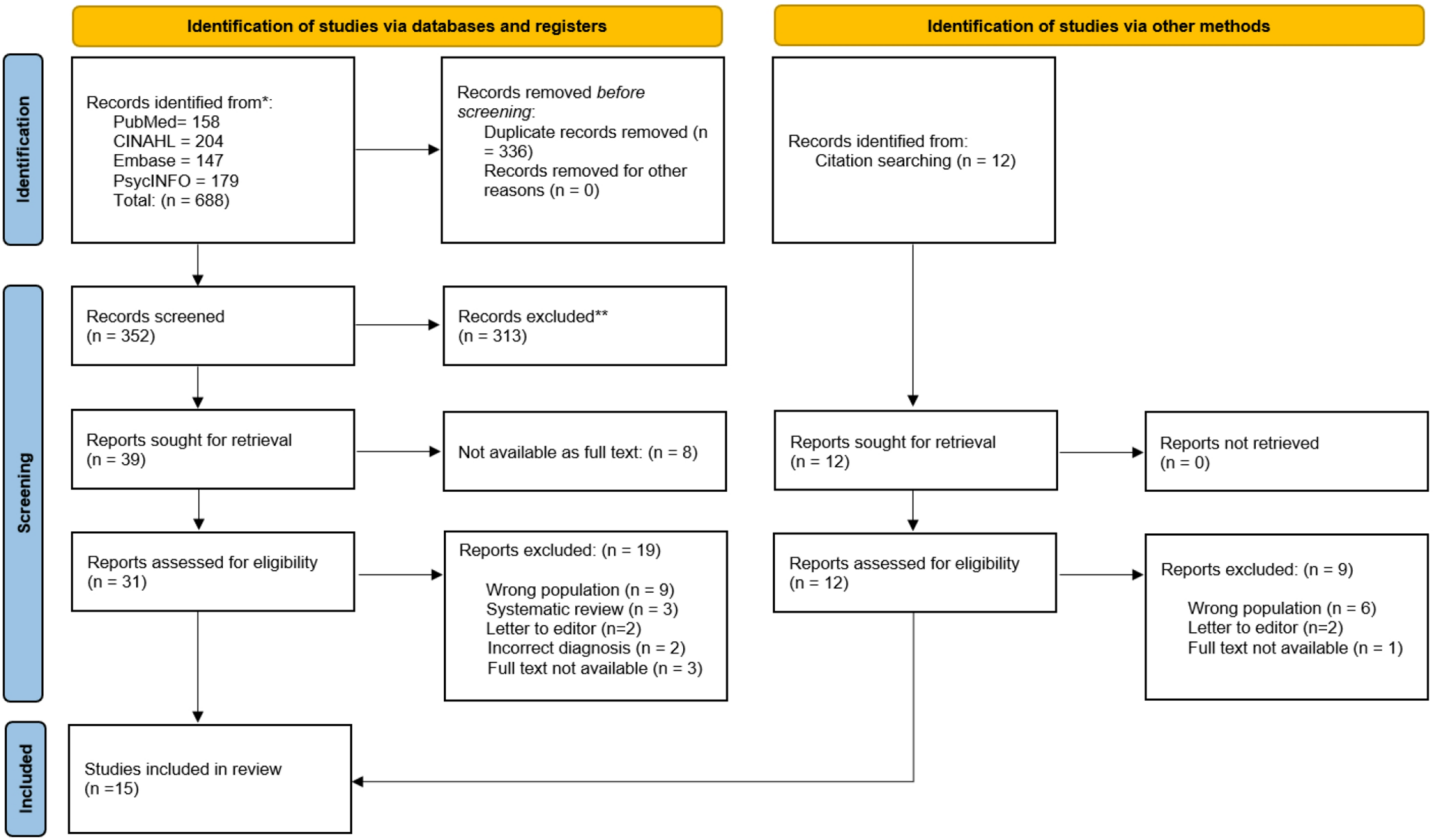
PRISMA flow diagram of study selection process [16]

Following duplicate removal, 352 studies were identified from electronic database searches. An additional 12 studies were identified through supplementary searching methods. Following title and abstract review of all studies, 43 full texts were reviewed in full. Fifteen studies were identified as meeting inclusion criteria. An overview of search results, including reasons for exclusion of studies, are contained in Fig. 1.

### Primary characteristics of included studies

Research representing the views of 2,591 clinicians from 26 countries was included across the fifteen eligible studies. Regions represented included Europe, North and South America, the Middle East, and Asia. Clinician views included those of: (1) consultant psychiatrists, (2) non-consultant hospital doctors specialising in psychiatry, (3) non-psychiatric specialist physicians, (4) advanced practice registered nurses, and (5) physician assistants. To elicit their perspectives on barriers and facilitators to clozapine prescribing, twelve studies used a survey design [19–29]. In two studies, semi-structured interviews were used [30, 31], or alternatively, a combination of quantitative and qualitative methods [32]. Table 1 summarises the key characteristics of the included studies and their key findings. In the case of barriers, due to their number and varying nature, these have been categorised, as in prior reviews [9], according to whether they relate to (1) clinicians, (2) patients, carers, and/or family members, or (3) institutional and healthcare systems.

**Table 1:** Key characteristics of included studies (listed in descending order by year of publication)

First author, year, title, study country*	Primary aim(s) objectives, methodology, study population	Key findings Barriers Facilitators	
Sarpatwari (2025) - Physician Experiences with and Perspectives on Clozapine Prescribing [29] USA	To understand physicians’ perceptions and experiences with the clozapine Risk Evaluation and Mitigation Strategy (REMS) program. Survey Psychiatrists (*n* = 165), Non-psychiatric specialist physicians (*n* = 31)	**Clinician-related**: Concern regarding side effects, particularly severe neutropenia **Patient-related**: Perception that blood tests are excessively burdensome **Healthcare system-related**: Administrative burden, including time-intensive nature of REMS process.	· Established structures facilitating improved patient-clinician conversations on clozapine treatment · Expanded clinician education to cover clozapine risks and effectiveness
Grant (2024) - Psychiatrists’ views on clozapine prescribing in Ireland [15]	Examine the knowledge of, attitudes towards and perceived barriers to clozapine use among consultant psychiatrists. Survey Consultant psychiatrists (*n* = 77)	**Clinician-related**: • Insufficient clinical experience • Lack of confidence in prescribing **Patient-related**: • Adherence to regular blood monitoring • Confidence in clozapine’s effectiveness • Compliance with inpatient initiation • Medical complications **Healthcare system-related**: Administrative burden	· Dedicated staff to facilitate clozapine initiation · Clinician education programs · Availability of point-of-care haematological testing · Less frequent haematological monitoring · Improved patient education · Audit of antipsychotic prescribing · Clozapine community initiation teams
Jakobsen (2023) - Non-prescribing of clozapine for outpatients with schizophrenia in real-world settings: The clinicians’ perspectives [32] Denmark	Explore case-specific clinician perceived reasons for non-clozapine prescribing. Survey and semi-structured qualitative interviews *Survey phase*: Psychiatrists (*n* = 13) Clinical care providers (*n* = 21) *Interview phase*: psychiatrists (*n* = 10)	**Clinician-related**: • Expected non-compliance with haematological monitoring • Concerns regarding side effects • Decompensation of mental health on switching • Concerns regarding clozapine efficacy **Patient-related**: Expected non-adherence **Healthcare system-related**: Clinician non-adherence to established clozapine guidelines	· Clinician education programmes · Availability of point-of-care electrocardiogram and haematological testing · Facilitated drug compliance and physical health monitoring · Audit and feedback on clozapine prescribing rates · Allocation of clozapine monitoring responsibilities among care providers
Rezaie (2023) - Exploration of the Barriers to Clozapine Prescribing in Patients with Treatment-Resistant Schizophrenia: A Qualitative Study [31] Iran	Explore psychiatrists’ point of view on the barriers to clozapine prescribing in patients with TRS. Semi-structured qualitative interviews Psychiatrists (*n* = 12)	**Clinician-related**: • Concerns about serious side effects • Requirement for additional physical health management and monitoring • Fear of legal repercussions in the event of agranulocytosis • Limited experience with clozapine prescribing **Patient and family-related**: • Low level of education • Low socioeconomic status • Lack of access to physical health monitoring facilities • Expected non-adherence **Health system-related**: • Lack of hospital beds for clozapine initiation • Complex initiation procedure • Administrative burden • Lack of standardised supports • Service fragmentation	· Clinician education programmes · Improved patient and family education · Allocated hospital beds for clozapine titration · Establishing follow-up and support teams · Improved availability of laboratory services
Rezaie (2022) - Iranian psychiatrists’ attitude towards clozapine use for patients with treatment-resistant schizophrenia: a nationwide survey [28]	Investigate the knowledge and attitude of Iranian psychiatrists towards clozapine use. Survey Psychiatrists (*n* = 282)	**Clinician-related**: • Concerns regarding side effects • Limited clinical experience **Patient-related**: • Cost of laboratory testing • Expected lack of agreement from patients and carers regarding treatment initiation and continued compliance **Healthcare system-related**: • Service fragmentation • Inadequate resourcing • Absence of follow up teams	· Improved clinician education and training · Clinician access to alternative clinicians experienced in clozapine prescribing · Patient and family education · Adherence to guidelines · Specialised clozapine clinics
Cotes (2022) - A Comparison of Attitudes, Comfort, and Knowledge of Clozapine Among Two Diverse Samples of US Psychiatrists [27]	Examine attitudes, training, knowledge and perceived barriers about clozapine among US psychiatrists. Survey Psychiatrists (*n* = 143)	**Clinician-related**: • Requirement for routine haematological monitoring • Concern regarding side effects • Limited prescribing experience **Patient-related**: • Expected non-adherence to clozapine • Expected patient concerns regarding side effects **Healthcare system-related**: • Administrative burden • Access to routine haematological monitoring	· Clinician education programmes · Specialised clozapine clinics · Audit and feedback of clozapine prescribing practices · Integration of psychiatric pharmacists into clozapine management services
Ristic (2021) - Prescription attitudes and practices regarding clozapine among Serbian psychiatrists: results of a nationwide survey [26]	Assess Serbian psychiatrists’ attitudes regarding obstacles to clozapine prescribing and their current prescription practices. Survey Psychiatrists (*n* = 161)	**Clinician-related**: Concerns regarding side effects **Patient-related**: Expected patient refusal to comply with haematological monitoring **Healthcare system-related**: • Absence of national monitoring guidelines • Absence of dedicated clozapine inpatient and outpatient facilities	· Access to clozapine serum assays · Clinician education programmes · Revised mandatory haematological monitoring · Availability of point-of-care haematological testing · Development and modification of guidelines · Establishment of a National Clozapine Expertise Centre
Hayek (2021), Prescribing Clozapine in the Middle East and North Africa (MENA) Region: The Perspective and Practice of Psychiatrists [25]	Survey psychiatrists in the MENA region on clozapine prescription patterns, practices, attitudes and perceived barriers to prescribing clozapine. Survey Psychiatrists (*n* = 245)	**Clinician-related**: • Requirement for laboratory monitoring • Concerns regarding haematological and metabolic side effects • Insufficient prescribing experience **Patient-related**: • Expected non-compliance with haematological monitoring and clozapine treatment • Expected fear of side effects • Management of drug-disease interactions • Cost of treatment • Poor social support **Healthcare system-related**: • Requirement for hospital admission • Insufficient staffing • Cost of inpatient treatment • Administrative burden	· Availability of regional and national guidelines · Dedicated clozapine clinics · Improved clinician education · Availability of point-of-care haematological testing · Improved patient and family education
Daod (2019) -Psychiatrists’ attitude towards the use of clozapine in the treatment of refractory schizophrenia: A nationwide survey [22] Israel	Examine the awareness, familiarity, and attitude of a nationwide sample of Israeli psychiatrists regarding clozapine use. Survey Psychiatrists (*n* = 295)	**Clinician-related**: • Insufficient knowledge of clozapine prescribing • Concerns regarding side effects • Concern regarding requirement for haematological monitoring **Patient-related**: • Burden of haematological monitoring • Cost of treatment • Expected non-adherence with clozapine • Drug-disease interactions **Healthcare system-related**: Availability of specialised clozapine resources associated with lower prescribing rates.	· Access to clozapine serum assays · Establishment of Clozapine National Support Information Centre to provide clinician guidance and education · Availability of point-of-care leukocyte testing
Singh (2020) - Comfort Level and Barriers to the Appropriate Use of Clozapine: A Preliminary Survey of US Psychiatric Residents [24]	Assess US psychiatry residents’ comfort levels prescribing clozapine and identify perceived barriers to the use of clozapine. Survey Psychiatric residents (*n* = 164)	**Clinician-related**: Concerns regarding side effects **Patient-related**: • Expected refusal to comply with haematological monitoring • Expected concerns regarding side effects • Patient lack of confidence in clozapine’s efficacy **Healthcare system-related**: • Limited access to dedicated clozapine clinics • Requirement for inpatient admission • Administrative burden	· Initial and continued training and supervision for clinicians. · Establishment of national teams of clinicians and researchers for the purposes of increasing access to clozapine. · Establishment of National Association of State Mental Health Program Directors (NASMHPD) to address barriers to clozapine accessibility.
Moody (2019) - Perceived Barriers and Facilitators of Clozapine Use: A National Survey of Veterans Affairs Prescribers [23] USA	Identify perceived barriers and facilitators to clozapine use within the Veterans Health Administration Survey Healthcare providers with clozapine prescribing credentials: Physician (*n* = 85) Advanced practice registered nurse (*n* = 9) Physician Assistant (*n* = 3)	**Clinician-related**: • Insufficient experience with prescribing clozapine • Concerns regarding side effects • Preference for alternative pharmacotherapies **Patient-related**: • Expected non-compliance with clozapine • Difficulties with transportation to monitoring and dispensing centres **Healthcare system-related**: • Intensive monitoring requirements including weekly absolute neutrophil count (ANC) for first six months • Administrative burden of REMS enrolment	· Dedicated clozapine clinics · Clinician clozapine education sessions · Availability of community-based titration · Nursing or pharmacy support for follow-ups during initiation
Tungaraza (2015) - Clozapine prescribing in the UK: views and experience of consultant psychiatrists [21]	Explore the views and experiences of consultant psychiatrists with regard clozapine use in the UK. Survey Psychiatrists (*n* = 243)	**Clinician-related**: • Concerns regarding side effects • Limited clinician experience and knowledge • Concerns regarding clozapine efficacy **Patient-related**: • Expected reluctance to adhere to haematological monitoring and clozapine treatment. • Poor social support **Healthcare system-related**: • Shortage of hospital beds • Service fragmentation • Poor community supports • Administrative burden	· Clinician education programs · Clozapine dedicated services including community-based titration
Grover (2015) - Prescription practices and attitude of psychiatrists towards clozapine: A survey of psychiatrists from India [19]	Assess the attitude of psychiatrists towards clozapine and evaluate clozapine prescribing practices in India. Survey Psychiatrists (*n* = 548)	**Clinician-related**: • Requirement for haematological monitoring • Concern regarding side effects • Insufficient experience and knowledge of clozapine prescribing • Preference for alternative pharmacotherapies, including polypharmacy **Patient-related**: • Expected non-adherence to haematological monitoring and clozapine treatment • Expected patient anxiety related to side effects • Cost of treatment • Poor social supports • Managing drug-disease interactions **Healthcare system-related**: Absence of dedicated clozapine clinics	· Improved clinician education on clozapine · Availability of side effect management guidelines
Silveira (2015) - Patterns of clozapine and other antipsychotics prescriptions in patients with treatment-resistant schizophrenia in community mental health centers in São Paulo, Brazil [20]	Evaluate the patterns of clozapine and other antipsychotic drugs prescription in TRS in community mental health centres. Survey Psychiatrists (*n* = 15)	**Clinician-related**: • Insufficient prescribing experience • Complex initiation procedure **Patient-related**: • Expected lack of patient adherence to haematological monitoring • Lack of family support • Expected patient anxiety regarding side effects • Potential for drug-disease interactions • Cost of treatment **Healthcare system-related**: • Laboratory delay and low reliability • Administrative burden • Limited availability of clozapine	· Governmental support and provision of medical training, equipment and logistic support · Improved blood count infrastructure · Clozapine cost reduction · Improved community access to clozapine
Nielsen (2010) - Psychiatrists’ attitude towards and knowledge of clozapine treatment [30] Denmark	Investigate the reasons for the delay and underutilisation of clozapine among Danish psychiatrists. Structured qualitative interviews Psychiatrists (*n* = 100)	**Clinician-related**: • Limited knowledge and experience of clozapine prescribing • Concerns regarding side effects **Patient-related**: • Perceived patient dissatisfaction with clozapine treatment • Expected patient refusal to adhere to haematological monitoring **Healthcare system-related**: Lack of education or promotion due to generic status of clozapine	· Dedicated clozapine clinics · Improved clinician education · Referral of clozapine eligible patients to specialised clinics by less experienced psychiatrists

*Country where study was completed was included where not obvious from study title

## Key findings

**Fig. 2:**
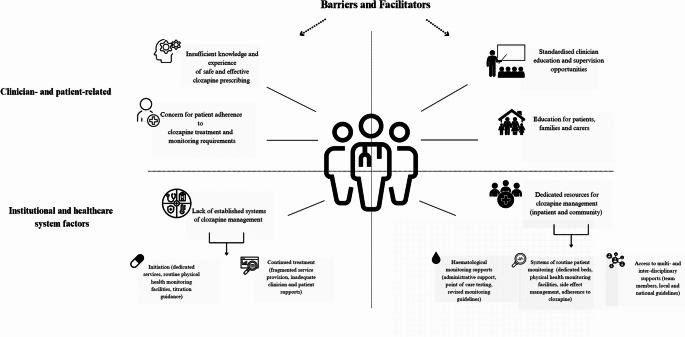
Overview of the most common barriers and facilitators to clozapine prescribing among clinicians’

Figure 2 provides an overview of the most commonly reported barriers and facilitators to clozapine prescribing by clinicians.

### Barriers to clozapine prescribing

1. **Clinician-related barriers**: Among the most common barriers to clozapine prescribing reported by clinicians internationally was limited knowledge and clinical experience of clozapine prescribing, particularly in relation to complex cases and rechallenge as reported in several studies [15, 19–32]. Concerns were reported regarding limited knowledge relating to clozapine’s initiation procedures [20–24, 28], monitoring protocols and procedures [19], side effect prevalence and management [15, 19, 21, 22, 26, 30], and management of drug-disease interactions among complex patient cohorts [15, 20, 22, 32]. Consequently, a lack of prescriber confidence in, and fear associated with, managing clozapine treatment were very common barriers reported [15, 19, 25, 29–31]. The side effects reported as most commonly inducing fear among clinicians included agranulocytosis [19–22, 24–26, 30, 31], myocarditis [15, 21–23, 25, 28–31], metabolic side effects [19, 21, 23–26, 32], seizures [15, 19, 21, 23–26, 29, 31], constipation [19, 21–23, 25, 26, 28], sedation [15, 19, 21, 23, 25, 26, 28, 32], and hypersalivation [19, 21, 23, 25, 28, 31]. In some countries, despite knowledge of its endorsed role within TRS management guidelines, clinicians also reported a lack of confidence in clozapine’s efficacy [21, 32] as well as concerns regarding decompensation of stable patients if switched to clozapine [32]. The requirement for routine haematological monitoring was another commonly cited barrier [15, 19–25, 27–31]. The administrative burden for prescribers and lack of confidence regarding patient adherence to routine haematological monitoring were the primary concerns among clinicians who cited haematological monitoring as a barrier to their increased prescribing [22, 25, 27, 30].
2. **Patient and family-related barriers**: Clinicians principally cited concerns about patients’ ability to consistently adhere to clozapine treatment and its physical health monitoring requirements as barriers to their prescribing [15, 20–23, 25–29, 31]. Other patient-related concerns included anticipated patient anxiety, reluctance, or refusal with clozapine treatment, including efficacy and side effect profile [15, 19–22, 24, 25, 28, 30, 31], ^,^ the cost of treatment [19, 20, 22, 28], and adequacy of patient supports and appropriate information for patients regarding clozapine treatment [19, 20, 25, 31].
3. **Institutional and healthcare system-related barriers**: The most common and significant institutional barrier to clozapine prescribing was the administrative burden on clinicians associated with initiating and continuing clozapine treatment [15, 20–25, 27, 29, 31]. Alongside this was the lack of established structures to support clinicians in initially establishing patients on clozapine treatment, and continuing this longitudinally [20, 21, 24, 25, 27, 31]. These included (i) a lack of dedicated inpatient beds to facilitate initiation [21, 24, 25], (ii) inadequate resourcing and dedicated staffing for clozapine management [25, 28], including physical health monitoring [23, 27], and (iii) the absence of community or stepdown systems of care to support shared care [24, 26, 28].

#### Facilitators of clozapine prescribing

Across all studies, clinicians reported the need for improved access to education and training in clozapine management as important facilitators to clozapine prescribing [15, 19–32]. As part of improved training initiatives, clinicians also highlighted the need for appropriate implementation supports, including access to clinicians more experienced in clozapine prescribing and experts in psychopharmacology, including psychiatric pharmacists [15, 23, 27, 28]. Availability of dedicated clozapine clinics, appropriately resourced with physical health monitoring and run by teams of multidisciplinary professionals experienced in clozapine management, was also a very commonly reported facilitator [15, 24, 25, 27, 30]. Access to haematological point-of-care testing was also reported as an important facilitator [15, 22, 25, 26, 32]. Less commonly reported facilitators included improved (i) patient and family education [15, 25, 28, 31], (ii) availability of clinical practice guidelines focussed on clozapine prescribing [19, 25, 26, 28], and (iii) availability of clozapine initiation services within the community [15, 20, 21, 23, 25]. Better audit and feedback of clozapine prescribing practices was also suggested by clinicians within a minority of studies [15, 27]. 

## Discussion

This systematic review represents the most contemporary understanding of barriers and facilitators to clozapine prescribing among clinicians within TRS management. Fifteen studies were included reflecting research conducted within many different settings, including countries not reflected within prior similar systematic reviews [9, 12, 13]. The review’s focused research question allows for targeted and evidence-based recommendations to be developed, informing what intervention strategies are necessary to increase clinician willingness to engage with clozapine treatment. Although some variation in local barriers was identified, particularly in developing countries, (e.g., the cost of clozapine in Brazil), the major barriers to clozapine prescribing included (1) limited knowledge and experience of clozapine prescribing, resulting in fear and lack of confidence associated with managing clozapine treatment, (2) concerns regarding patient suitability for clozapine treatment, including their ability to continually adhere to clozapine and its monitoring requirements, and (3) the lack of established structural supports within specialist and community settings to both safely initiate and continue clozapine treatment [23, 27, 28]. 

The most commonly reported facilitators included (1) improved and standardised access to targeted training and educational supports, alongside opportunities for supervision by more experienced colleagues, (2) increased availability of supports designed to reduce the administrative burden among clinicians when initiating clozapine treatment, and (3) dedicated structures, including access to multidisciplinary teams and physical health monitoring resources, that support prescribers in achieving safe, effective and guideline-adherent clozapine treatment. The latter includes facilitating patient adherence to haematological and metabolic monitoring, effectively managing side effects of clozapine, and supporting both continued patient adherence to clozapine and engagement with effective psychiatric treatment [20, 28, 30, 31]. 

### Mastering the art of clozapine treatment – addressing clinician fear associated with prescribing clozapine

From this review, and as identified previously [9], lack of knowledge regarding clozapine’s role as a first-line treatment in the management of TRS is not a major barrier to its prescription, although lack of confidence in its superiority and concern for destabilisation were reported within a small number of studies [25, 26]. Instead, a fear of clozapine (so-called “*Clozaphobia*”) [33], appears a universal barrier. Among clinicians internationally, factors identified as contributing to a fear of clozapine treatment included concerns about (and misconceptions about the rate of) serious side effects, lack of prescribing experience and an absence of standardised supports in safely managing clozapine treatment. The perceived complexity of clozapine prescribing, including its distinctive initiation and reinitiation procedures [33], and concern for decompensation on switching pharmacotherapies [7], are also factors identified as increasing clinician fear associated with prescribing clozapine.

The “*art of clozapine therapy*” has previously been described [34]. In comparison to other antipsychotics, clozapine has a unique pharmacological profile [35]. Clozapine binds to a broad range of distinct receptors outside of its supposed target muscarinic acetylcholine receptors. This includes various subtypes of serotonin, histamine and alpha-adrenergic receptors – both inside and outside of the central nervous system [35]. Consequently, clozapine also has a distinct side-effect profile, particularly in relation to its potential impacts on physical health parameters. Many of these side effects have the potential to be serious and extend beyond well-known risk of agranulocytosis. These include side effects relating to respiratory (e.g., aspiration pneumonia), cardiac (myocarditis, cardiomyopathy) and gastrointestinal (e.g., clozapine-induced gastrointestinal hypomotility, ileus) systems [2]. The perceived complexity of clozapine prescribing and the factors contributing to the clinician fear of managing clozapine treatment requires purposeful intervention.

Improved availability of clinical practice guidelines was suggested by clinicians within several settings as a facilitator of improved clozapine prescribing [25, 26, 28]. Suggestions for the content of these guidelines included those addressing side effect management and practice recommendations that have both regional and national relevance. Similar to the recent INTEGRATE (International guidelines for the algorithmic treatment of schizophrenia) guidelines [36], harmonized international guidelines may reduce both fear of clozapine prescribing among clinicians and reduce some aspects of clinician administrative burden by providing ease of access to reliable, evidence-based and internationally-relevant prescribing and monitoring [37]. 

Whilst some prior guidelines addressing clozapine prescribing and side effect management are available [38], a recent systematic review identified variable quality in guideline development and called for future development processes to align with established international standards [38]. Such guidelines will, however, also require appropriate implementation supports. Based on facilitators identified within this review, this should include improved and standardised access to supervision by colleagues experienced in clozapine management. The impact of improved availability of high-quality guidelines compared to increased practical experience with prescribing clozapine, particularly early in clinical training, on increasing the rate of clozapine prescribing is worthy of future research.

### Clozapine treatment as a burden among prescribers – the need for structural interventions

Aside from fear of clozapine treatment, perhaps the most significant barrier to clozapine prescribing is the perceived burden its prescription and continued management represents. Review findings indicate that clinicians consider the burden of clozapine prescribing on themselves, patients and families, and healthcare institutions as a barrier to prescribing. The perceived burden of clozapine treatment is typically paired with (1) unclear treatment benefits to patients secondary to a lack of clinician confidence in patients’ likelihood to adhere to treatment and monitoring [15, 19–23, 26, 27, 30–32], and (2) a lack of appropriate facilitative structures within healthcare systems to support clinicians achieving safe and effective initial and continued treatment [15, 20, 22–31]. Clinicians often reported assumptions they made on behalf of patients regarding their personal assessment of the burden of clozapine treatment on them and made judgements regarding this burden outweighing potential benefits [14, 19]. Such assumptions were often heightened where clinicians’ perceived patients to have poor social supports, including supportive family members [19, 21, 25]. The most commonly reported facilitators reported by clinicians to prescribing clozapine focused on reducing the perceived burden of clozapine treatment, particularly on their own practice. This typically included establishing specialist teams and services dedicated to initiating and maintaining patients on longitudinal clozapine treatment.

To date, most interventions designed to improve rates of clozapine prescribing have targeted individual prescribers, for example, through use of local audit and feedback cycles or local guidelines [9, 39]. However, results of this review indicate that most barriers and facilitators exist in realms beyond the influence of individual prescribers. Furthermore, whilst one important barrier, review results confirm that additional interventions beyond recent relaxation in haematological monitoring, including recent measures by the USA’s Federal Drug Agency [40], and the European Medicines Agency [41], will be required. To systematically increase rates of clozapine prescribing, structural interventions will instead be needed. These interventions should focus on modifying the healthcare contexts in which TRS management and clozapine prescribing occurs [10]. Necessary components of these interventions include (1) establishing appropriately resourced teams with expertise in psychopharmacology and clozapine management, (2) resources to facilitate patient adherence clozapine and required physical health monitoring parameters, including metabolic and haematological monitoring, and (3) access to interdisciplinary supports, including cardiology or haematology expertise, to support the management of more complex cases, or in cases of significant drug-disease interactions. Furthermore, studying established healthcare systems that have achieved and sustained high rates of clozapine prescribing would complement review results and provide an understanding of what unique elements of associated intervention strategies are effective at increasing clinician prescribing rates [17]. For example, in London, the Maudsley Treatment Review and Assessment Team (TREAT) was established to facilitate community management of TRS [42]. Assessments of the effectiveness of this service included a five-fold increase in the rate of clozapine initiation and may serve as a model for other countries internationally [42]. 

Review results should be considered in the context of several potential limitations. First, risk of bias assessments of individual studies, and a similar assessment of the certainty of evidence across the totality of evidence, were not conducted. Correspondingly, equal weighting of evidence quality was given to all studies. Formal assessment of the quality of individual studies may have changed this approach, and thus, review conclusions. Conducting such assessments should be the focus of future reviews, for example, using the GRADE (Grading of Recommendations Assessment, Development and Evaluation) system [43]. Second, as most of the included studies used survey methodology, as in all surveys, results are limited by the potential for selection bias, where those inherently interested in, or knowledgeable regarding, clozapine prescribing, may be more likely to respond [9]. Third, whilst the case was made for focusing the review question on barriers and facilitators among clinicians responsible for prescribing clozapine, patient, family and carer views also require due consideration and should be the focus of a future, updated systematic review. Finally, barriers and facilitators to increase clozapine prescribing may be different within adolescent or older adult populations. As most people presenting with TRS are not represented by these populations [1], we excluded studies that solely focused on these populations. Recommendations offered here may be different outside of the populations included in this review.

## Conclusion

Among clinicians responsible for prescribing clozapine, major barriers reported included (i) clinician-related barriers, (ii) patient- and carer-related barriers, and (iii) institutional and healthcare system barriers. Across all three categories, major barriers involved (i) a lack of confidence, and fear, associated with prescribing clozapine, (ii) the often personal and significant administrative burden its management represents, and (iii) the lack of established systems of care to support safe and effective longitudinal treatment with clozapine. Facilitators reported included increased access to education and supervision opportunities and establishing dedicated and appropriately resourced clozapine clinics across both inpatient and community settings. Thus, most of the barriers and facilitators reported by clinicians as being essential to their prescribing of clozapine are beyond the influence of individual prescribers. Consequently, much greater organisational and institutional responsibility will be required to increase clinician engagement. Structural interventions that focus on modifying the contexts in which healthcare delivery is provided are necessary and should be a priority among those responsible for designing and funding mental health services. Such interventions will be essential in increasing equitable patient access to clozapine. Of primary importance within such interventions are increasing the supports and expertise available to individual prescribers. Increased supports for prescribers are needed at the point of clozapine initiation, during monitoring of response to treatment, when managing side effects common to clozapine, and in facilitating continued patient adherence with treatment.

## Supplementary Information

Below is the link to the electronic supplementary material.

Supplementary Material 1 (DOCX 36.2 KB)

## Data Availability

Data availability is not applicable to this article as no new data were created or analysed in this study.
